# High-Discrimination Circular Polarization Detection Based on Dielectric-Metal-Hybrid Chiral Metamirror Integrated Quantum Well Infrared Photodetectors

**DOI:** 10.3390/s23010168

**Published:** 2022-12-24

**Authors:** Jinyong Shen, Tianyun Zhu, Jing Zhou, Zeshi Chu, Xiansong Ren, Jie Deng, Xu Dai, Fangzhe Li, Bo Wang, Xiaoshuang Chen, Wei Lu

**Affiliations:** 1 State Key Laboratory of Infrared Physics, Shanghai Institute of Technical Physics, Chinese Academy of Sciences, Shanghai 200083, China; 2 Hangzhou Institute for Advanced Study, University of Chinese Academy of Sciences, Hangzhou 310024, China; 3 University of Chinese Academy of Sciences, Beijing 100049, China; 4 Shanghai Institute of Optics and Fine Mechanics, Chinese Academy of Sciences, Shanghai 201800, China

**Keywords:** monolithic circular polarization detection, dielectric-metal-hybrid chiral metamaterials, quantum well infrared photodetector, high circular polarization extinction ratio, absorption efficiency enhancement

## Abstract

Circular polarization detection enables a wide range of applications. With the miniaturization of optoelectronic systems, integrated circular polarization detectors with native sensitivity to the spin state of light have become highly sought after. The key issues with this type of device are its low circular polarization extinction ratios (CPERs) and reduced responsivities. Metallic two-dimensional chiral metamaterials have been integrated with detection materials for filterless circular polarization detection. However, the CPERs of such devices are typically below five, and the light absorption in the detection materials is hardly enhanced and is even sometimes reduced. Here, we propose to sandwich multiple quantum wells between a dielectric two-dimensional chiral metamaterial and a metal grating to obtain both a high CPER and a photoresponse enhancement. The dielectric-metal-hybrid chiral metamirror integrated quantum well infrared photodetector (QWIP) exhibits a CPER as high as 100 in the long wave infrared range, exceeding all reported CPERs for integrated circular polarization detectors. The absorption efficiency of this device reaches 54%, which is 17 times higher than that of a standard 45° edge facet coupled device. The circular polarization discrimination is attributed to the interference between the principle-polarization radiation and the cross-polarization radiation of the chiral structure during multiple reflections and the structure-material double polarization selection. The enhanced absorption efficiency is due to the excitation of a surface plasmon polariton wave. The dielectric-metal-hybrid chiral mirror structure is compatible with QWIP focal plane arrays.

## 1. Introduction

The polarization of light [1,2,3,4] contains important information. In addition to linear polarization detection, circular polarization detection provides a new dimension of polarization information, and thus enables a wide range of applications such as anti-jamming imaging [5], chiral molecule screening [6], and high-fidelity communication [7,8,9]. With the miniaturization of optoelectronic devices and systems [10,11,12,13,14,15,16,17,18,19], filterless integrated circular polarization detectors are sought to replace traditional devices relying on separate polarizers and wave plates. Flat optics have been proposed to substitute for conventional polarizers and wave plates to reduce the sizes of circular polarization detectors [20,21,22,23,24]. However, the sophisticated alignment during heterogeneous integration and the reduction in light flux received by the photosensitive regions hinder the implementation of flat optics for polarization detector miniaturization. Thus, more and more attention has been concentrated on filterless detectors with native circular polarization discrimination. The most straightforward idea for designing spin-sensitive photodetectors is to use chiral detection molecules with their own circular polarization selectivity [25,26,27,28]. However, the circular polarization discrimination of these materials is usually low, and the response band of the material is very limited, resulting in a narrow selection range. With the development of micro-nano technology, integrating chiral metamaterial with detection materials for circular polarization dependent light coupling becomes a potential route. Three-dimensional (3D) chiral metamaterials, such as helical metamaterials [29,30,31,32,33,34,35], can induce high CPERs. The CPER is defined as the ratio of the absorption efficiency under the illumination of circularly polarized light with the designated handedness to the absorption efficiency under the illumination of circularly polarized light with the opposite handedness. However, they are difficult to fabricate and integrate with infrared detection materials. The recently developed nano-kirigami method based on one-step focused ion beam fabrication seems to be a way to create 3D chiral metamaterials with giant circular dichroism [36]. At the same time, two-dimensional (2D) chiral metamaterials [10,11,12,37,38], which can be easily fabricated and integrated with active materials, attract a lot of research interest. It is worth mentioning that a recently developed plasmonic diatomic metasurface exhibits a sharp differentiation of spectral responses for an arbitrary pair of orthogonal polarization states [37]. Unfortunately, they only induce low CPERs (typically below two) for light absorption in active materials, and limited absorptances of active materials due to severe ohmic loss and impedance mismatch. Recently, chiral metamirrors have been widely investigated for circular dichroic absorption [11,39,40,41,42], because they induce higher CPERs (typically three to five) than 2D chiral metamaterials and involve much easier fabrication processes than three-dimensional chiral metamaterials. A chiral metamirror usually consists of a 2D chiral metamaterial layer, a dielectric spacer, and a metal plane. The thickness of the dielectric spacer can be adjusted for impedance matching. The circularly polarized light (CPL) with designated handedness is efficiently coupled into a light mode mainly confined within the spacer, and the CPL with the opposite handedness is mostly reflected. When the spacer is replaced by a detection material, a prominent circular-polarization-discriminative absorption in the detection material is achieved. However, the ohmic loss of the metal parts impedes light absorption in the detection materials, although an impedance matching condition could be reached by tuning the thickness of the detection material [39,41,42,43,44,45]. We recently revealed that integrating an anisotropic detection material with a chiral metamirror can further enhance the CPERs to 14 based on the double polarization selection provided by the chiral metamirror and the detection material [40]. However, the CPERs of chiral metamirror integrated circular polarization detectors are still far lower than those of traditional circular polarization detectors with separated polarizers and wave plates. It can be seen that the key issues affecting integrated circular polarization detectors are their low CPERs and reduced responsivities.

In this work, we address these key issues by integrating a dielectric-metal-hybrid chiral metamirror with a quantum well infrared photodetector (QWIP) for both a high CPER and a high absorption efficiency. The multiple quantum wells are sandwiched between a dielectric two-dimensional chiral metamaterial and a metallic reflective grating. The CPER of this device is as high as 100 in the long wave infrared range, exceeding all reported CPERs for integrated circular polarization detectors. The absorption efficiency of this device [46], i.e., the absorptance of the quantum wells, reaches 54%, which is 17 times higher than that of the same quantum wells in a standard 45° edge facet coupled device. In addition, the peak CPER of our device remains above 48 within the incident angle range of ±10°, indicating that the QWIP is compatible with an optical system with an F number larger than 2.8, and the dielectric-metal-hybrid chiral metamirror structure is compatible with QWIP focal plane arrays. Thus, the dielectric-metal-hybrid chiral metamirror integrated QWIP is expected to be a promising solution for infrared circular polarization detection.

## 2. Materials and Methods

**Figure 1:**
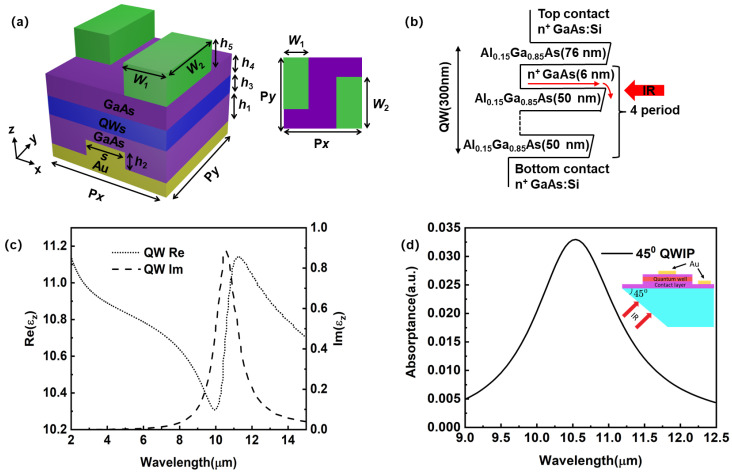
(**a**) Schematic diagram of the 3D structure of the dielectric-metal-hybrid chiral metamirror integrated quantum well infrared photodetector (QWIP). The inset figure shows the schematic diagram of a *Z*-groove unit cell in the *x*-*y* view. Structural parameters: *P_x_* = 3.9 μm, *P_y_* =3.9 μm, *W*_1_ = 1.25 μm, *W*_2_ = 2.5 μm. The thicknesses of the bottom contact layer, the grating, the QWs, the uncarved part of top contact layer, and the 2D chiral structure are *h*_1_ = 560 nm, *h*_2_ = 360 nm, *h*_3_ = 300 nm, *h*_4_ = 520 nm, *h*_5_ = 4.35 μm, and *s* = 1.326 μm. The chiral metamaterial is drawn in green. (**b**) The active region with a total thickness (*h*_3_) of 300 nm contains four AlGaAs (50 nm)/GaAs (6 nm) QW stacks. (**c**) Real and imaginary parts of *ε_z_*. The dielectric constant of the effective medium of the QWs is diag (*ε_x_*, *ε_y_*, *ε_z_*). (**d**) Absorption efficiency of a standard 45° edge facet coupled device.

Figure 1a shows the structure of the dielectric-metal-hybrid chiral metamirror integrated QWIP. The dielectric-metal-hybrid chiral metamirror is periodic in the *x*-*y* plane. The multiple III-V semiconductor layers are grown by epitaxy and integrated with the dielectric-metal-hybrid chiral metamirror in a flip-chip manner, which is generally used by focal plane arrays [40,44,47,48,49]. The bottom contact layer made of heavily doped GaAs has a thickness (*h*_1_) of 560 nm. The reflective grating is carved out of the bottom contact layer with a depth (*h*_2_) of 360 nm. The metal ridge width (*s*) of the grating is 1.326 μm. The active region with a total thickness (*h*_3_) of 300 nm contains 4 AlGaAs (50 nm)/GaAs (6 nm) quantum well (QW) stacks in Figure 1b. The top GaAs contact layer has a thickness (*h*_4_) of 520 nm. The dielectric 2D chiral metamaterial on the top of the circular polarization detector is shown in Figure 1a of the inset figure. The 2D chiral metamaterial has a two-dimensional periodic structure within the *x*-*y* plane. The period along the *x*-axis (*P_x_*) and that along the *y*-axis (*P_y_*) are both 3.9 μm. In a single period, there are two GaAs cuboids of the same size, forming a *Z*-shape groove. The geometry of the *Z* groove is defined by the following parameters: *W*_1_ = 1.25 μm, *W*_2_ = 2.5 μm, *h*_5_ = 4.35 μm. The chiral metamaterial can be carved out of the top GaAs contact layer by etching. Due to the selection rule of the intersubband transition, the QWs only absorb the light with a *z*-component electric field. Thus, the QWs can be regarded as an uni-axial effective medium with a permittivity tensor like *ε*_QW_ = diag (*ε_x_*, *ε_y_*, *ε_z_*), where *ε_x_* = *ε_y_* = *ε*_GaAs_ and $\varepsilon_{z}=\varepsilon_{GaAs}+\frac{\varepsilon_{GaAs}f_{12}\omega_{p}^{2}}{\omega_{12}^{2}-\omega^{2}-i\omega \gamma }$ [50]. In the *x*-*y* plane, the optical response of the QWs is similar to that of GaAs, which is a transparent dielectric in the long wave infrared range. In the *z*-direction, *ε_z_* conforms to the Lorentz formula. ε_GaAs_ is the refractive index of the GaAs, *f*_12_ the oscillator strength, *ω*_p_ the two-dimensional effective plasma frequency, *ω*_12_ the transition frequency in optics, and *γ* the relaxation frequency [51]. Figure 1c shows Re (*ε_z_*) and Im (*ε_z_*) of the QWs in a typical LWIR QWIP with the peak detection wavelength of 10.55 μm. Since the QWs do not respond to normally incident light, they are usually made into a 45° edge facet coupled device for photoresponse characterization [46]. The absorptance of the QWs in the 45° edge facet coupled mode is considered as a standard reference. Figure 1d shows the absorption efficiency spectra of a standard 45° edge facet coupled device. At 10.55 μm, the absorption peak is 3.3%. The dielectric constant of Au is derived from the Drude model [52].

## 3. Results and Discussion

**Figure 2:**
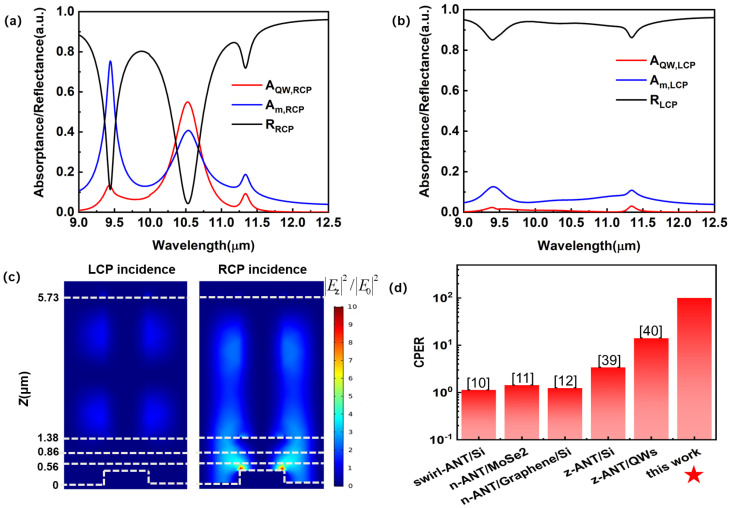
(**a**) Absorptance and reflectance spectra of the dielectric-metal-hybrid chiral metamirror integrated QWIP under RCP illumination. A_QW, RCP_ denotes the absorptance of the QWs, A_m, RCP_ denotes the absorptance of the metal, R_RCP_ denotes the reflectance of RCP. (**b**) Absorptance and re-flectance spectra of the dielectric-metal-hybrid chiral metamirror integrated QWIP under LCP illu-mination. A_QW, LCP_ denotes the absorptance of the QWs, A_m, LCP_ denotes the absorptance of the metal, R_LCP_ denotes the reflectance of LCP. (**c**) Field distributions on the *x*-*z* cross section of the dielectric-metal-hybrid chiral metamirror integrated QWIP under LCP and RCP illumination, respectively. The wavelength of the incident light is 10.55 µm. The colored surfaces represent |*E_z_*|^2^/|*E*_0_|^2^. (**d**) Comparison of between the CPER of our device and the reported CPERs of different integrated circularly polarized detectors.

Figure 2a,b show the absorptance and reflectance spectra of a dielectric-metal-hybrid chiral metamirror integrated QWIP under LCP and RCP illumination. RCP light induces a prominent resonance at the wavelength of 10.55 μm, where the absorption efficiency of the device reaches 54%. The prominent resonance at the wavelength of 10.55 μm corresponds to a surface plasmon polariton (SPP) wave at the interface between the metal grating and the bottom GaAs contact layer (Figure 2c). The metal grating provides an extra tangential wave vector equal to the propagation constant of the SPP wave. Accordingly, the normally incident light with an electric component along the *x*-direction could effectively excite the SPP wave. When the SPP mode is excited, an intensified local field is built up and both the absorptance of the QWs and that of the metal are increased, resulting in a reflectance as low as 5%. In comparison, LCP light does not effectively excite the SPP wave, and it is largely reflected by the dielectric-metal-hybrid chiral metamirror integrated QWIP. The discrimination of the handedness of the light is due to the interference between the principle-polarization radiation and the cross-polarization radiation of the chiral structure during multiple reflections. In brief, if we consider the dielectric-metal chiral metamirror as a film for interference, the SPP waves excited by LCP light during the multiple reflections in this film destructively interfere with each other, while, in contrast, the SPP waves excited by RCP light during the multiple reflections constructively interfere with each other. The multiple-reflection interference is analyzed in detail later. Further, the circular polarization discrimination is magnified by the double polarization selection provided by the chiral metamirror and the QWs [19,40,45]. Since the QWs only absorb the light with a z-component electric field, the SPP wave excited by the RCP is efficiently absorbed by the QWs, while the LCP light directly incident on the QWs and that diffracted or scattered into the QWs is hardly absorbed, leading to CPER magnification. Thus, LCP light is highly reflected by the device over the LWIR range from 9 to 12.5 μm. The absorptance of the QWs is fairly low. Especially at the peak detection wavelength of the QWs (10.55 μm), the absorption efficiency is only 0.54%, and the CPER at this wavelength is as high as 100. As shown in Figure 2d, the CPER of 100 achieved by the dielectric-metal-hybrid chiral metamirror integrated QWIP significantly exceeds the reported CPERs of integrated circular polarization detectors [10,11,12,39,40]. Before this work, the combination of a chiral metamirror and an anisotropic detection material (e.g., QWs) exhibited great potential for achieving high CPERs. In comparison, the dielectric-metal-hybrid chiral metamirror integrated QWIP proposed in this work enhances CPERs significantly more. Compared with the CPER of an ordinary chiral metamirror integrated QWIP [40], the CPER of the dielectric-metal-hybrid chiral metamirror integrated QWIP is more than seven times higher. In addition to the higher CPER, our device also has the advantages of a lower ohmic loss and a higher absorption efficiency. For a typical chiral metamirror made of two metal layers sandwiching a dielectric layer, the SPP wave usually causes a considerable ohmic loss. For a chiral metamirror integrated QWIP such as the one we studied previously [40], 46% of the incident power is absorbed by the metal parts at the SPP resonance excited by the CPL with the designated handedness, and the absorption efficiency is 36%. In this work, since we replace the top metal layer with a dielectric layer, the ohmic loss is reduced to 40%, and the absorption efficiency is increased to 54%. The 54% absorption efficiency is 17 times higher than that of the standard 45° edge facet coupled device in Figure 1d, indicating that the dielectric-metal-hybrid chiral metamirror integrated QWIP not only has a high circular polarization discrimination but also has an enhanced absorption efficiency.

**Figure 3:**
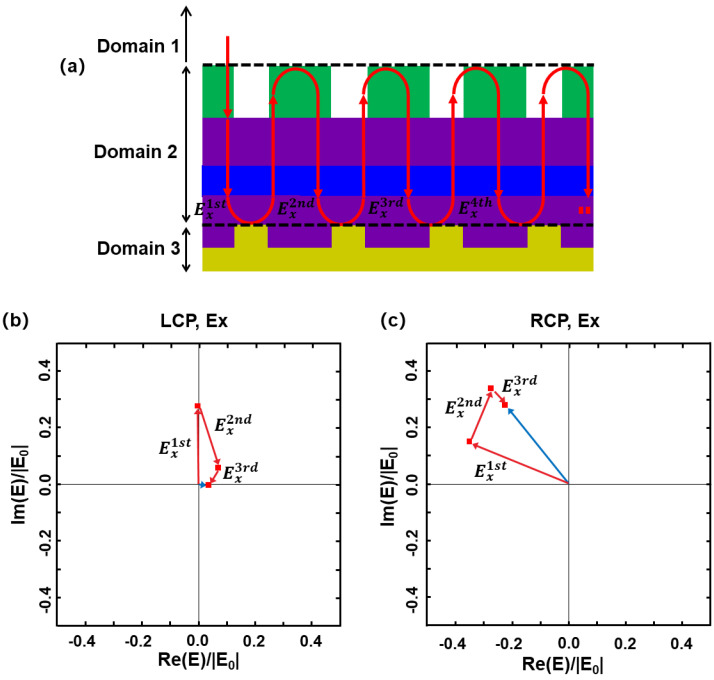
(**a**) Sketch of multiple reflections in the dielectric-metal-hybrid chiral metamirror inte-grated QWIP. (**b**) or (**c**) Evolution of the light field (*E_x_*) impinging at the metal grating after being reflected at the semiconductor/grating interface and the semiconductor/air interface for the 1st, 2nd, and 3rd time under LCP or RCP illumination.

The circular polarization discrimination mechanism is revealed by the multiple-reflection interference picture, as sketched in Figure 3a. In this picture, the space is divided into three domains: air, semiconductor layer, and metal grating. The incident light from the air passes through the dielectric chiral structure layer, propagates in the semiconductor layer (GaAs/QWs/GaAs), and impinges on the grating. A part of the light power excites the SPP wave, and the rest is reflected back. Then, the reflected light bounces back and forth between the semiconductor/air interface and the semiconductor/grating interface and excites SPP waves multiple times. All of these SPP waves interfere with each other, leading to the final field strength of the SPP mode. The light incident at the grating for the nth time may be written as

(1) $$E=\sum_{n}{\left[e^{i2k_{a}h}r_{21}r_{23}\right]}^{n-1}e^{ik_{a}h}t_{12}E_{0}$$

$E_{0}=\left(E_{0x},E_{0y}\right)$ denotes the incident light from the air; $t_{12}$ the transmission coefficient at the air/semiconductor interface; *k_a_* the wave vector in the semiconductor layer; *h* the effective thickness of the semiconductor layer; $r_{23}$ the reflection coefficient at the semiconductor/grating interface; and $r_{21}$ the reflection coefficient at the semiconductor/air interface. *h* takes a value of 1.22 μm, smaller than the real height of the semiconductor layer including the *Z*-groove region, since the *Z*-groove region is less dense than GaAs. *n* is an integer that is not smaller than 1. $t_{12}$, $r_{23}$, and $r_{21}$ are second order tensors. $r_{23}$ is a diagonal tensor since the metal grating is a symmetric structure. $t_{12}$ and $r_{21}$ both contain non-diagonal elements due to the chiral structure. These non-diagonal elements correspond to cross-polarization radiation, and lead to a discrimination between RCP and LCP light. The discrimination is magnified after multiple reflections. This process is demonstrated by tracing the light field impinging at the metal grating after different numbers of round trips, as shown in Figure 3b,c. Since the SPP wave can only be excited by the *x*-component of the electric field (*E_x_*), we only plot the evolution of *E_x_* for RCP and LCP light. *E_x_* is plotted as a vector in a complex coordinate system, so the superposition of the *E_x_* fields during the multiple-reflection process is represented by the superposition of the vectors. It is interesting to note that the amplitude of $E_{x}^{1st}$ for RCP light is similar to that for LCP light, indicating that both RCP and LCP light could excite the SPP wave when they are first incident on the metal grating. The discrimination occurs when the light is incident on the metal grating for the second time after a round trip between the semiconductor/grating interface and the semiconductor/air interface. For RCP light, $E_{x}^{2nd}$ constructively interferes with $E_{x}^{1st}$ to some extent, and then $\left|E_{x}^{1st}+E_{x}^{2nd}\right|>\left|E_{x}^{1st}\right|$ (Figure 3b). For LCP light, $E_{x}^{2nd}$ destructively interferes with $E_{x}^{1st}$, and $\left|E_{x}^{1st}+E_{x}^{2nd}\right|$ is much smaller than $\left|E_{x}^{1st}\right|$ (Figure 3c). After more round trips, the total *E_x_* for RCP almost has the same amplitude as $E_{x}^{1st}$, while, in contrast, the total *E_x_* for LCP dramatically reduces due to destructively interference. As a result, the SPP wave is prominently excited by RCP light, while it is not effectively excited by LCP light, leading to significant circular polarization discrimination. As demonstrated in Figure 3b,c, after three reflection processes, ${\left|E_{x}^{1st}+E_{x}^{2nd}+E_{x}^{3rd}\right|}_{\mathrm{RCP}}^{2}$ is 106 times larger than ${\left|E_{x}^{1st}+E_{x}^{2nd}+E_{x}^{3rd}\right|}_{\mathrm{LCP}}^{2}$.

**Figure 4:**
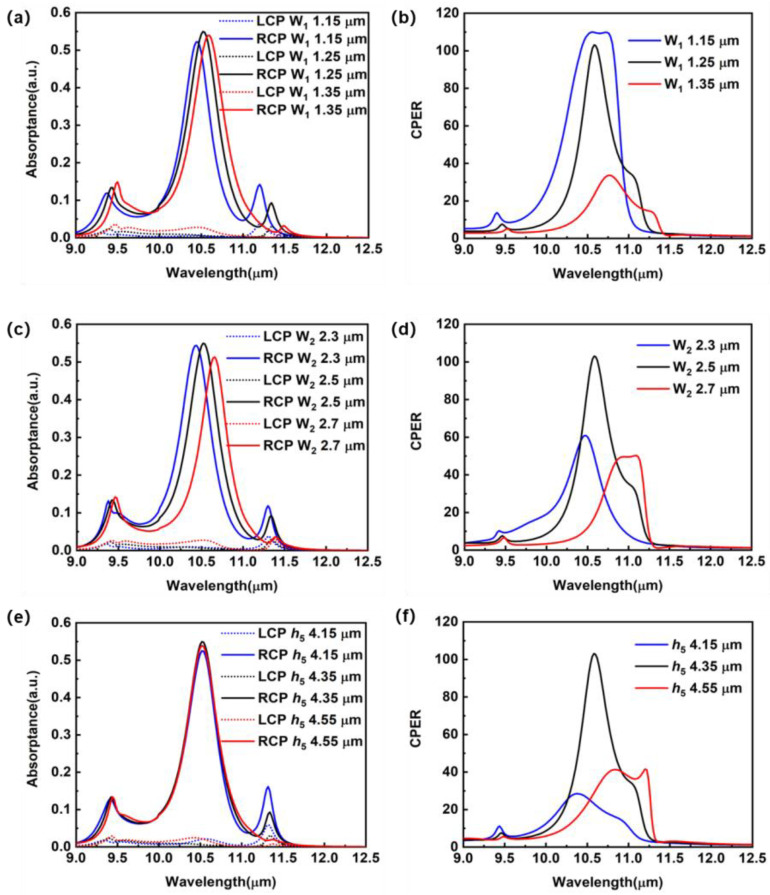
(**a**) Absorption efficiency spectra of the dielectric-metal-hybrid chiral metamirror integrated QWIPs with different *W*_1_ values under RCP and LCP illumination. The rest of the geometrical parameters remain the same as for the device presented in Figure 1. (**b**) CPERs corresponding to the different *W*_1_ values. (**c**) Absorption efficiency spectra of the devices with different *W*_2_ values under RCP and LCP illumination. The other geometrical parameters remain the same as for the device presented in Figure 1. (**d**) CPERs corresponding to the different *W*_2_ values. (**e**) Absorption efficiency spectra of the devices with different *h*_5_ values under RCP and LCP illumination. The rest geometrical parameters remain the same as the device presented in Figure 1. (**f**) CPERs corresponding to the different *h*_5_ values.

The influence of the *Z*-groove geometry on the circular polarization dependent photoresponse is presented in Figure 4. The basic geometry is the optimized geometry (*W*_1_ = 1.25 μm, *W*_2_ = 2.5 μm, *h*_5_ = 4.35 μm) that we presented in the previous context. Then, we change only one parameter at a time. As shown in Figure 4a, with the increasing *W*_1_, the absorption peak induced by the SPP wave is red-shifted. This is due to the fact that the increase in *W*_1_ enlarges the proportion of GaAs in the *Z*-groove layer and thus enlarges the effective refractive index of the whole semiconductor layer. Concerning the circular polarization discrimination, a smaller *W*_1_ such as 1.15 μm slightly improves the CPERs to 110 (Figure 4b), but reduces the LCP light induced absorptance of the QWs to 43% at the designated wavelength (10.55 μm) due to the SPP resonance shift. Increasing *W*_1_ to 1.35 μm substantially reduces the CPER to 21 at the wavelength of 10.55 μm. Concerning the influence of *W*_2_, the increase in *W*_2_ red-shifts the SPP resonance (Figure 4c) just like the increase in *W*_1_ based on the same reason. In addition, either an increase or a decrease in *W*_2_ induces substantial drop in the CPER (Figure 4d). As shown in Figure 4e, the absorption peak due to the SPP wave excited by RCP light is slightly affected by the thickness of *Z*-groove. However, for LCP, the deviation of *h*_5_ from the optimized value weakens the destructive interference of the SPP wave, leading to a substantial drop in the CPER (Figure 4f).

**Figure 5:**
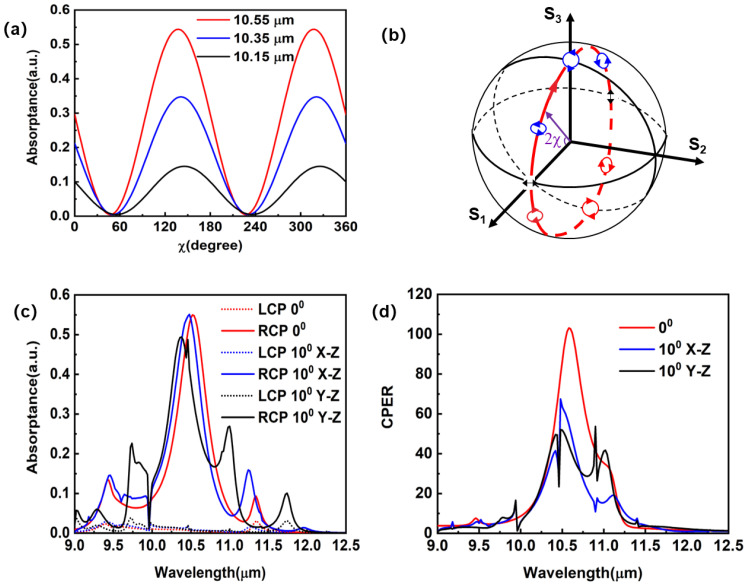
(**a**) Light ellipticity angle dependent absorption efficiency of the dielectric-metal-hybrid chiral metamirror integrated QWIP at different wavelengths. (**b**) Poincare sphere with a longitude circle crossing the *S*_1_ axis. (**c**) Absorption efficiency spectra for LCP and RCP light at an oblique incidence of 10° in the *x*-*z* and *y*-*z* plane. (**d**) CPER spectra for oblique incidence of 10° in the *x*-*z* and *y*-*z* plane.

The photoresponse of the dielectric-metal-hybrid chiral metamirror integrated QWIP to the light in other polarization states has also been investigated. Figure 5a shows the photoresponses versus the light ellipticity angle (*χ*) at three wavelengths. The orientation angle (*ψ*) is fixed at 0°. With the varying *χ*, the polarization state moves along a longitude line on the Poincare sphere [53] (Figure 5b). As *χ* increases from 0° to 180°, the polarization state of the incident light goes through linear polarization along the *x*-direction (*χ* = 0°), left circular polarization (*χ* = 45°), linear polarization along the *y*-direction (*χ* = 90°), and right circular polarization (*χ* = 135°), before returning to the original state (*χ* = 180°). The polarization states in the upper hemisphere are left-handed polarization states, and those in the lower hemisphere are right-handed ones. As shown in Figure 5a, at χ = 45° (LCP), the absorptance of the QWs reaches a minimum. At χ = 135° (RCP), the absorptance of the QWs reaches a maximum. It is worth noting that the absorptance of the QWs at χ = 0° (linear polarization along the *x*-direction) is different from that at χ = 90° (linear polarization along the *y*-direction). This is attributed to the fact that the absorptance induced by the asymmetric structure (*Z* groove) depends on the linear polarization angle.

The angle dependence of the circular polarization detector is another important property, because, in practical applications, the light reaching the detector after passing through an optical system covers a certain range of incident angles. As shown in Figure 5c, at the oblique incidence of 10° in the *x*-*z* plane, the SPP resonance is slightly blue-shifted. The absorption efficiency for RCP light becomes 48% at the wavelength of 10.55 μm. For LCP light, the oblique incidence ruins the destructive interference among the multiple reflections, and thus the suppression of the absorptance becomes less effective. As shown in Figure 5d, the CPER of the device with an obliquely incident light at 10° in the *x*-*z* plane reduces to 59. Similarly, the oblique incidence of 10° in the *y*-*z* plane also blue-shifts the SPP resonance, and thus results in an absorption efficiency of 32% at the wavelength of 10.55 μm. The oblique incidence in the *y*-*z* plane also increases the absorption efficiency for LCP, so the CPER is reduced to 48. Although an oblique incidence of 10° reduces the performance of the dielectric-metal-hybrid chiral metamirror integrated QWIP, the absorption efficiency for the principle circular polarization (higher than 32%) together with the CPER (higher than 48) still exceeds the performances of most integrated circular polarization detectors, indicating that the QWIP is compatible with an optical system with an F number larger than 2.8.

## 4. Conclusions

In summary, we proposed a dielectric-metal-hybrid chiral metamirror integrated QWIP for both high CPER and high absorption efficiency. Based on the interference between the principle-polarization radiation and the cross-polarization radiation of the chiral structure during multiple reflections, and the structure-material double polarization selection, the CPER of our device reaches 100. The excitation of the SPP wave improves the absorption efficiency to 54%, which is 17 times higher than that of the standard 45° edge facet coupled device. In addition, the peak CPER of our device remains above 48 within the incident angle range of ±10°, indicating that the device can be practically used in an optical system with an F number larger than 2.8. Further, the architecture of the dielectric-metal-hybrid chiral metamirror integrated QWIP is compatible with focal plane arrays. Our work provides a promising route for the development of high-performance monolithic circular polarization detectors.

## Data Availability

Not applicable.
